# Sleep-Wake Cycle and Daytime Sleepiness in the Myotonic Dystrophies

**DOI:** 10.1155/2013/692026

**Published:** 2013-11-04

**Authors:** A. Romigi, M. Albanese, C. Liguori, F. Placidi, M. G. Marciani, R. Massa

**Affiliations:** ^1^University of Rome “Tor Vergata”, Policlinico Tor Vergata, Department of Systems Medicine, Section of Neurophysiopathology, Sleep and Epilepsy Centre, 00133 Rome, Italy; ^2^University of Rome “Tor Vergata”, Policlinico Tor Vergata, Department of Systems Medicine, Section of Neurosciences, 00133 Rome, Italy; ^3^Fondazione S. Lucia IRCCS, 00100 Rome, Italy

## Abstract

Myotonic dystrophy is the most common type of muscular dystrophy in adults and is characterized by progressive myopathy, myotonia, and multiorgan involvement. Two genetically distinct entities have been identified, myotonic dystrophy type 1 (DM1 or Steinert's Disease) and myotonic dystrophy type 2 (DM2). Myotonic dystrophies are strongly associated with sleep dysfunction. Sleep disturbances in DM1 are common and include sleep-disordered breathing (SDB), periodic limb movements (PLMS), central hypersomnia, and REM sleep dysregulation (high REM density and narcoleptic-like phenotype). Interestingly, drowsiness in DM1 seems to be due to a central dysfunction of sleep-wake regulation more than SDB. To date, little is known regarding the occurrence of sleep disorders in DM2. SDB (obstructive and central apnoea), REM sleep without atonia, and restless legs syndrome have been described. Further polysomnographic, controlled studies are strongly needed, particularly in DM2, in order to clarify the role of sleep disorders in the myotonic dystrophies.

## 1. Introduction

Myotonic dystrophies are autosomal, dominantly inherited, progressive, and multisystemic disorders characterized by neuromuscular weakness, myotonia, early-onset cataracts, endocrine abnormalities, and involvement of other organs including CNS, heart, and gastrointestinal system [1, 2].

There are 2 clinically, histopathologically, and genetically distinct forms of myotonic dystrophy: type1 (DM1) and type2 (DM2), although a high degree of clinical overlap exists between these two entities [3, 4].

DM1 is the most common form of muscular dystrophy in adults, with a prevalence ranging from 5 to 12 per 100000 population among Caucasians. Based on age at onset and severity of symptoms, 4 clinical types of DM1 can be distinguished: mild, adult-onset, early childhood, and congenital onset. The phenotype and genetics of DM2 have been described more recently, and its prevalence has not yet been clearly established. 

Sleep disturbances are well described in neuromuscular diseases. The presence of sleep dysfunction is also a major cause of morbidity and mortality in patients with neuromuscular disorders. Persistent nocturnal hypoxemia, the end result of sleep disordered breathing (SDB) from any cause, results in cardiovascular and pulmonary failure; in addition, sleep fragmentation and excessive daytime sleepiness lead to disability and may affect mood and cognition.

DM1 represents the neuromuscular disorder with the most prominent propensity to sleep disorders and excessive daytime sleepiness [5]. SDB, resulting in nocturnal hypoxia and hypercapnia, periodic limb movements of sleep (PLMS), REM sleep dysregulation, and daytime somnolence seem to be the most common sleep disorders in patients with DM1 [6–8]. Several lines of evidence suggest that DM1 may have direct effects on sleep regulatory circuits in the CNS as demonstrated by the loss of serotoninergic neurons of dorsal raphe nucleus and low cerebrospinal fluid (CSF) orexin A levels [9, 10]. These patients may also exhibit signs of central sleep-wake regulation dysfunction, including excessive daytime sleepiness out of proportion to SDB, fatigue, and abnormalities of REM sleep [6–8, 11]. Scarce data are available regarding sleep disorders in DM2, due to the more recent genetic definition and to the rarity of the disease in some geographic areas [12]. DM2, similar to DM1, may be characterized by SDB and sleepiness [13–15] and REM sleep dysregulation [15], albeit few, and uncontrolled polysomnographic data are available. Here, we review the extant literature in order to stimulate discussion that may eventually lead to better understand the real magnitude of sleep dysfunction in myotonic dystrophies, improve clinical care of these patients, and aid in their clinical distinction.

## 2. Genetic Features of Myotonic Dystrophies

While DM1 results from expansion of a CTG trinucleotide repeat in the *DMPK* gene on chromosome 19q13.3, a CCTG repeat expansion in the *CNBP* (*also known as ZNF9*) gene located on chromosome 3q21.3 and encoding for a ubiquitous protein has been identified in DM2 [16, 17]. Both mutations lead to formation of transcript aggregates in the nucleus, so-called foci, which interfere with proteins that play a part in RNA metabolism, including members of the muscleblind (MBNL) family of RNA-binding proteins. Dysregulation of these proteins (due to sequestration or inappropriate phosphorylation) results in missplicing of several downstream effector genes and causes loss of function of gene products, which are thought to account, at least in part, for multiorgan involvement/multisystemic phenotype in DM [18]. 

Unlike DM1, in which increased repeat length causes the phenomenon of genetic anticipation (thereby influencing disease severity and causing earlier symptom development and presentation in successive generations), in DM2, repeat length does not seem to correlate with disease severity and does not show anticipation [1, 2, 19]. 

Despite similarities in their molecular pathology, myotonic dystrophy types 1 and 2 are clearly different disorders. Distinctive clinical features in patients with DM2 include the propensity for less severity, proximal rather than distal weakness, and lack of prominent muscle wasting. In addition, several key features of DM1, including facial weakness, ptosis, and respiratory insufficiency, and a congenital form, are typically absent in DM2. Nevertheless, differentiation between the two myotonic dystrophies on clinical grounds alone is often difficult, and genetic evaluation is necessary to make a definitive diagnosis [1–3, 16].

## 3. Daytime Somnolence in Myotonic Dystrophy: Its Prevalence and Pathophysiology

Excessive daytime sleepiness (EDS) is the most common nonmuscular symptom in DM1, which occurs in up to 70–80% of adult-onset DM1 [20] and in ~50% of childhood-onset DM1 patients [21]. Several studies demonstrated that EDS may be an early DM1 symptom, appearing sometimes even years before the disease is recognized [22–24].

Unlike in narcolepsy, EDS in DM1 is characterized by persistent sleepiness unaffected by naps, the latter being frequently long, unrefreshing, and without dream content. In addition, sleepiness mainly occurs in monotonous situations or when attention is not being held [25–27] and its complaint is mostly unrelated to the duration and the quality of the nocturnal sleep [11, 28]. 

Although in several studies DM1-related EDS is strongly associated with obstructive, central sleep apnoea and/or hypoventilation [22, 29, 30], other authors reported the lack of correlation between EDS and sleep apnoea in DM1 [10, 23, 24, 31, 32]. On the other hand, a neuropathological study showed a selective loss of serotoninergic neurons of dorsal raphe nucleus, a key region involved in sleep-wake modulation, in DM1 patients complaining of EDS [9]. Furthermore, low CSF levels of orexin A were described in hypersomnolent DM1 patients similarly to narcolepsy [10], albeit this finding was not successively confirmed [33]. In addition, *narcoleptic-like* patients were also described in DM1 [31, 34]. Hypersomnia and DM1 are frequently associated with the HLA haplotype DRW6-DQW1, different from those frequently observed in the narcoleptic or nonnarcoleptic types of hypersomnia [10, 33–35]. Finally, modafinil resulted effective in DM1-related EDS [36]. Therefore, a “central” origin of hypersomnolence in DM1 was conjectured.

## 4. Daytime Sleepiness: Clinical Features and Diagnostic Assessment

Some studies suggested that DM1 patients with EDS are younger and have an earlier age at disease onset and a greater muscular impairment than patients without EDS, without differences for gender, body mass index (BMI), and the number of CTG repeat. They are also more prone to psychological distress, tending to be less sociable, optimistic, active, empathetic, persistent, organized, and motivated in goal-directed behaviors when compared with those without EDS [37–39].

As a general rule, EDS is firstly and mainly mentioned by close relatives. It results as a prominent and debilitating clinical feature of the disease, being rarely reported spontaneously by patients themselves [37, 40, 41]. 

Besides EDS, fatigue is more common in DM1 than in other neuromuscular disorders and may even be significant when muscular impairment is relatively mild [42]. DM1-related fatigue is characterized by a subjective lack of physical and/or mental energy, partially improved by rest, and is present in up to 74% of adult-onset DM1 patients [28]. 

Since EDS and fatigue are associated in DM1 patients and share overlapping features, both patients and physicians may often have difficulties distinguishing EDS from fatigue [28, 42]. Patients may be unable to specify whether their complaint relates to sleepiness, fatigue, or both. 

Apathy may also represent a confounding factor, because it may be confused with EDS. Furthermore, its characteristics and clinical correlates remain unclear [43]. However, one study using standardized rating scales reported that these symptoms are independent features of the disease, an issue that needs replication and further investigation [24]. 

Diagnostic assessment of EDS is performed by means of some standardized tests which quantify objective daytime sleepiness. Unfortunately, these techniques require sophisticated equipment, sleep training and are expensive and highly time consuming. The Multiple Sleep Latency Test (MSLT) and the Maintenance of Wakefulness Test (MWT) remain the gold standard for assessment of EDS in both general and DM1 populations [5, 7]. While the MSLT mainly measures physiological degrees of sleepiness in relation to the sleep drive [44], the MWT may provide a more sensitive indicator of the variation of alertness in relation to the wake drive in DM1 [45]. Other objective methods for monitoring vigilance, including the behavioral OSLER test [46] and the 10-minute Psychomotor Vigilance Test-192 [47], should be more systematically tested in the DM1 patients in order to validate their clinical reliability. 

Although few studies reported a pathological mean sleep latency (MSL) on the MSLT as defined by an MSL ≤ 8 min in DM1 patients [10, 31] with a positive correlation with the degree of muscular impairment [6], most studies did not find a positive relationship between daytime sleepiness complaints and MSLT results [11, 37, 48]. In the same line of evidence, Romigi et al. demonstrated a significantly lower MSL in DM1 versus controls suggesting a natural “propensity” of DM1 to somnolence without reaching pathological levels of EDS [7]. 

Moreover, the subjective evaluation of daytime sleepiness and fatigue levels may be based on clinical interview and self-reported questionnaires. These instruments may represent a useful tool, albeit disease-specific validated scales should be necessary in order to better quantify these specific symptoms. Sleepiness is not a unitary notion and may be responsible of different states and/or symptoms. Therefore, several assessment tools for EDS have been developed; the majority of them offer useful data, but they do not grasp all aspects of sleepiness particularly when applied in specific diseases with symptoms resembling sleepiness (i.e., fatigue, apathy) [49]. A previous study of reliability suggested a weak internal consistency for the Epworth Sleepiness Scale (ESS) in patients with DM1, rendering its current usage in DM1 questionable [43, 50]. One alternative tool, the Daytime Sleepiness Scale (DSS), consists of 5 items that are closer to the real life of DM1 patients and to the clinical features most commonly complained in association with DM1-related EDS (i.e., daytime napping and sleepiness). DSS scores positively related to the extent of muscular impairment [32, 50]. 

Fatigue rating scales such as the Krupp's Fatigue Severity Scale (KFSS), which are based on the behavioral consequences of fatigue, may also constitute a more accurate and comprehensive measure of fatigue severity in the DM1 population [50]. 

Finally, there is still debate about the reliability between MSLT/MWT and subjective rating scales (either ESS and DSS). Some authors underlined lacking or poor correlation [7, 11, 28, 33, 37], suggesting that these “*clinical*” measures may explore different components of sleepiness in DM1 and may result in appropriate tools to evaluate the potential impact of somnolence on quality of life of DM1 patients [41]. 

Very recently, the Rasch-built Fatigue and Daytime Sleepiness Scale (FDSS), specifically utilized in DM1 patients, provided interval values on a single continuum between fatigue and sleepiness [51]. Therefore, these clinical instruments should be used for future clinical trials and therapeutic follow-up.

## 5. Nocturnal Sleep: Measurements, Sleep Architecture, and REM Sleep Dysregulation

DM1 patients report more sleep complaints than control subjects. A nonrestorative sleep and diurnal tiredness are often reported in several studies [6, 7, 32, 50]. In addition, habitual bedtime, wake-time, and time in bed were not different in DM1 with or without diurnal drowsiness [7, 37]. High variability of nocturnal sleep quality and quantity with either disrupted [26, 52, 53] or deep and restorative sleep [22, 52, 54] has also been repeatedly documented in DM1 patients by means of subjective and objective instruments. This sleep phenotype, with long and nonrestorative sleep during the night and the diurnal sleep episodes, is particularly frequent in DM1, being close to that of idiopathic hypersomnia with long sleep time [11]. Although many authors described a longer nighttime sleep in DM1 patients than in control subjects with a nonrestorative nocturnal sleep [32, 54, 55], this observation was not always confirmed [6, 7, 11, 22, 34, 54]. The lack of a control group may have affected some of these studies precluding a comparative evaluation as regards sleep diary, polysomnography (PSG), and MSLT measures [11, 31]. 

Other patients with DM1 reported a nighttime sleep fragmentation related to respiratory events, nocturnal agitation, reduced motility in bed, and pain [11]. In particular, DM1 patients with fatigue and EDS more often experienced diurnal and nocturnal feelings of pain due to back pain, headache, arthritis, and cramps, in comparison with patients without fatigue and EDS [8]. Pain probably has a multifactorial etiology and may be related to the peripheral vascular symptoms such as coldness and episodic pallor of hands and feet commonly referred [27]. 

To date, only a few and contradictory studies have focused on the nocturnal sleep continuity and sleep architecture of DM1 patients in comparison with control subjects by means of polysomnographic data. Some authors described normal sleep architecture and similar total sleep time in patients and controls, but others reported longer objective nocturnal sleep duration [22, 48, 52]. 

A prospective case-control study has revealed similar total sleep time and sleep efficiency but a decrease in percentage of stage 2 NREM sleep, a higher percentage of slow-wave sleep and REM sleep in DM1 patients than in controls, and an increased microarousal index [6].

In a recent controlled study, Romigi et al. demonstrated a significant reduction in total sleep time and sleep efficiency together with a significant increase in Wakefulness After Sleep Onset (WASO) and in stage 1 NREM in DM1 patients [7], confirming the presence of a disrupted sleep and abnormal sleep macrostructure found in previous uncontrolled studies [26, 30, 59]. 

Even though daytime sleepiness may be mainly attributable to sleep fragmentation, rare studies compared sleep parameters of DM1 patients with and without EDS and concluded that PSG findings were largely similar in terms of sleep continuity (sleep latency, total sleep time, and sleep efficiency) and sleep architecture [6, 7, 11].

Rapid eye movement (REM) sleep dysregulation has been also detailed in DM1 [8]. The presence of sleep-onset REM periods (SOREMPs) on the MSLT is a relatively frequent finding in DM1 as well as short sleep latencies, suggesting a *narcoleptic-like* phenotype and hypocretin CSF deficiency in DM1 supports this view [10]. Previous uncontrolled studies reported that DM1 patients recruited on the basis of EDS complaints presented at least 2 SOREMPs in 33% to 60% of cases [23, 31]. Otherwise, among DM1 populations unselected for EDS, the proportion of patients with one or more SOREMPs ranged from 25% to 50% [6, 11, 33]. Also, DM1 patients with several SOREMPs were noted to be younger and to show lower daytime sleep and nighttime REM sleep latencies, milder hypoxemic condition, and higher muscular impairment in comparison with DM1 patients without any SOREMP [6, 33].

Other REM sleep abnormalities include a higher REM density in DM1 patients with twofold increase compared to controls and a tendency for higher percentage of REM sleep without atonia (RSWA) and REM sleep phasic EMG activity in the absence of clinically assessed REM behavior disorder [6]. Although this finding may be of uncertain clinical significance, it may confirm the hypothesis of a “central” REM sleep dysregulation in DM1 [8].

## 6. Sleep Disorders in DM1: Sleep-Disordered Breathing (SDB)

SDB represent the most common cause of sleep dysfunction in neuromuscular diseases. Therefore, SDB is also frequent and well described in DM1, resulting in nocturnal hypoxemia, nocturnal hypoventilation, obstructive apnoeas, and hypopneas as well as central apnoeas [11, 30, 52, 56, 60]. SDB may be explained by both the involvement of respiratory muscles (weakness and myotonia) and abnormalities of central control of ventilation.

SDB may cause transient nocturnal hypoxemia and episodes of desaturation associated with microarousals and sleep fragmentation that may trigger EDS. However, strong evidence indicates that EDS in DM1 patients may occur in the absence of any identified respiratory events [6, 11, 22, 31] and, conversely, that DM1 patients may exhibit clinically significant SDB in the absence of EDS, snoring, and apnoea [30, 52, 61]. Recently, a controlled polysomnographic study showed a more impaired nocturnal sleep, as expressed by means of lower total sleep time and sleep efficiency and higher WASO and REM sleep latency, in DM1 with obstructive sleep apnoea when compared to DM1 with PLMS and without any sleep disorders [7]. 

Also, no relationship was noted between daytime pulmonary function tests results and subjective or objective daytime sleepiness [11, 55]. 

In a series of 43 DM1 patients unselected for EDS complaints, it was found that patients with pathological MSL on the MSLT had more severe respiratory muscle weakness, lung volume restriction, and daytime hypercapnia. Yet, Apnoea-hypopnoea index (AHI) was significantly correlated with total lung capacity and vital capacity, but these lung volume measurements accounted for only 16% of AHI variance [11]. Most authors believe that respiratory muscle weakness alone does not provide an adequate explanation for abnormalities of breathing during sleep in DM1 [6]. This hypothesis is supported by findings of severe neuronal loss in various medullary nuclei linked to respiratory function in DM1 patients with alveolar hypoventilation [62]. However, hypercapnia does not necessarily relate to EDS, since DM1 patients without CO_2_ retention may exhibit EDS [30, 52]. Inversely, correction of the alveolar hypoventilation rarely leads to the elimination of EDS [56]. Finally, the alveolar hypoventilation participates in the production of EDS only in a minority of cases [27].

## 7. Sleep Disorders in DM1: Restless Legs Syndrome & Periodic Limb Movement

Restless Leg Syndrome (RLS) and PLMS were also described in DM1 [6, 7, 21, 33]. 

Laberge et al. reported mean PLM indexes in wake (PLMW) and in sleep (PLMS) at 5.3/h and 10.5/h, respectively, in DM1 [11]. Subsequently, a case-control study confirmed these high rates (>50% of PLMS index above 5 and 40% above 10) in both NREM and REM sleep and especially in patients also presenting RLS [6]. Romigi et al. described PLMS (defined as PLMI > 5) as the most represented sleep disturbances (61.1%) in DM1 patients compared with controls [7]. In particular, these authors observed a highly significant increase in PLM index, which may represent a polysomnographic marker of DM1, confirming the data obtained in childhood-onset DM1 [21]. Altogether, these studies failed to report any relationship between PLMW/PLMS, arousability, and drowsiness. Hence, the clinical significance of PLMS remains unclear. A clinical phenotype ranging from narcoleptic symptoms to asymptomatic PLMS may be the result of a dysfunction of dopamine pathways in DM1. The high number of PLMS observed in DM1 may be a manifestation of premature ageing of the CNS, in line with the evidence of brain degenerative mechanisms in DM1 [63]. In addition, PLMS may represent a further puzzling finding for their potential link with an increased autonomic-related cardiovascular risk [64, 65]. Sleep disorders in DM1 are summarised in Table 1.

## 8. Pathophysiology of Sleep Dysfunction in DM1

DM1 is a progressive multisystemic disorder with a major brain involvement [66]. EDS is a frequent and severe clinical feature of DM1, but its pathogenesis remains conjectural. EDS of DM1 patients assessed by both objective instruments and self-report questionnaires is often out of proportion to their sleep disruption. Also, there is no coherent link between PSG abnormalities and evidence of SDB. Altogether, current data stress the hypothesis that there is a primary central disorder of arousal responsible for EDS intrinsic to DM1 disease pathology [55, 67]. Many patients with DM1 present metabolic-endocrine abnormalities, in particular dysfunction of the Hypothalamic-pituitary-adrenal (HPA) axis with a decreased pulsatile secretion of cortisol and growth hormone (GH) that may affect sleep regulatory circuits [27, 68]. Abnormal proinflammatory and hypnogenic cytokines secretion with increased levels of interleukin-6 and tumor-necrosis factor-alpha were reported in DM1 when compared with controls and may be related to sleepiness and fatigue [69]. A brain/brainstem disorder leading to altered sleep-wake cycle with central hypersomnia and REM sleep dysregulation may also be posited in DM1 [70]. Some authors have found a high incidence of intracytoplasmic inclusions in the anterior and dorsomedial thalamic nuclei with a loss of neurons and gliosis in the reticular formation, the midbrain, and pontine raphe in patients with DM1 together with a lower density of serotonin-containing neurons in the raphe [9, 62]. These findings may explain the presence of sleep dysfunction, EDS, apathy, and mental decline in DM1. On the other hand, cortical atrophy, dilatation of ventricles, and supratentorial bilateral symmetrical focal or diffuse white matter hyperintense lesions, either a specific or typical in the temporoinsular regions, have been well detected in DM1 patients by several conventional MRI studies [71]. While some authors showed correlations of brain morphological changes with neuropsychological and clinical parameters including CTG repeat sizes in DM1 [71], others failed to do so [57, 63]. Minnerop et al. confirmed white matter alterations in the whole brain, brainstem, and corpus callosum in association with grey matter decrease in cortical areas, thalamus, putamen and global cerebral atrophy, using voxel-based morphometry analysis and diffusion tensor imaging [66]. Both depressed mood and fatigue or increased daytime sleepiness were more pronounced in DM1 patients with less prominent white matter affection and at early disease stages, suggesting a reactive adjustment disorder instead of a direct consequence of structural brain damage [66]. Interestingly, few PET and SPECT studies documented a significant reduction of glucose metabolism and/or blood perfusion in DM1 patients compared to controls, mostly involving the left cortical areas and especially the frontal lobe. These metabolic changes seem to be proportional to the overall (clinical and genetic) severity of the disease and to explain selective alteration of executive functions better than the structural abnormalities revealed by conventional MRI [71]. Nevertheless, it is unclear whether these phenomena are due to a defect in vascular extraction and/or in membrane flux of the adopted tracers (“membrane disease” interpretation) or more easily related to synaptic protein dysregulation and synaptic dysfunction as recently theorized in transgenic animal models (“toxin RNA” paradigm) [58]. 

As DM1 patients share some REM sleep dysregulation with *narcoleptic-like* phenotype, several studies focused on a possible involvement of both the HLA haplotypes and hypocretin system in the disease [10, 33, 35]. However, available data do not report any association between DM1, particular HLA haplotypes, and CSF hypocretin-1 levels even in selected DM1 patients with complaint of EDS and high daytime REM sleep propensity.

## 9. Narcoleptic-Like Phenotype in DM1: A Case Report

**Figure 1:**
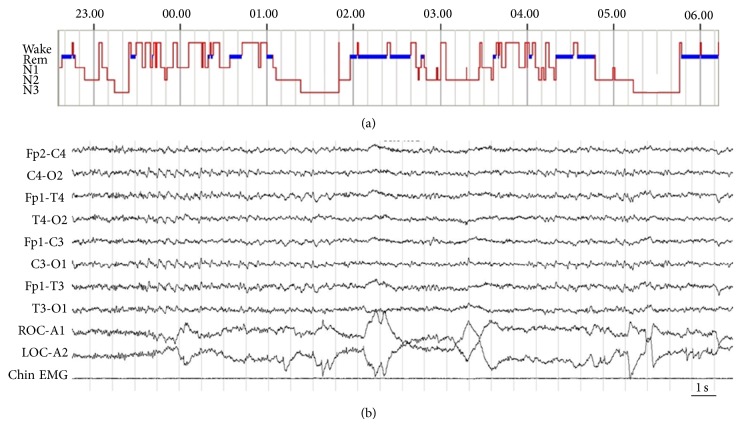
Sleep features of a DM1 patient with narcoleptic-like phenotype. (a) Narcoleptic-like hypnogram. Notice the short sleep onset latency, early onset of REM sleep, frequent nocturnal arousals, and REM sleep dysregulation. (b) A 30 s epoch of MSLT of the same patient demonstrating sleep onset REM sleep (SOREMPs). This patient showed 3 SOREMPs in a 4-nap protocol MSLT. ROC & LOC: right and left electrooculogram. F4, F3, C3, C4, T4, T3, O1, and O2: electrode positions according to the 10/20 International System. Chin: electromyography of the mentalis muscle.

A 40-year old man diagnosed with adult-onset DM1 referred nonrefreshing sleep, excessive daytime sleepiness with falling asleep inappropriately during the daytime despite averaging 8 hours of sleep per night, and nightmares. In the previous years, he noted increasing postprandial drowsiness and an irresistible urge to fall asleep ranging from 5 to 6 times daily. He also complained of hypnagogic hallucinations and some episodes of body paralysis in which he awakened unable to move for a few seconds (sleep paralysis) but did not report episodes of attacks of muscle atonia triggered by strong emotions (cataplexy). His medical history was unremarkable; he was not obese (BMI 27 kg/m^2^) and showed an Epworth Sleepiness Scale score of 16. On the basis of these clinical data, he underwent a PSG study, which showed an increased REM sleep time, which appeared as the 34% of the total sleep time, and a sleep efficiency of 81.2% with an AHI of 8.9. His sleep architecture was unusual in that sleep onset was slightly less than normal (few seconds) followed by an immediate REM sleep onset (2 min) (see Figure 1(a)). The MLST was performed after PSG and showed a mean sleep latency of 4.25 minutes with 3 SOREMPs (see Figure 1(b)). The human leukocyte antigen (HLA) haplotype determination did not show the “narcoleptic” DQB1∗06:02 haplotype, a rare association in hypersomnolent narcoleptic-like DM1 patients [33, 34]. Finally, a brain MRI was performed and appeared unremarkable. Therefore, considering the overall clinical history, the polysomnographic results, a diagnosis of DM1-related narcoleptic-like phenotype was made and the patient started treatment for hypersomnolence with Modafinil 200 mg/day, with a good response. Although DM1 is frequently associated with severe EDS and SOREMPs, similar to those observed in narcolepsy, several groups failed to determine an association with the “narcoleptic” HLA haplotype and with low CSF hypocretin-1 levels [8]. In addition, DM1 with drowsiness and HLA DQB1∗0602 are not associated as in narcolepsy, but a higher frequency of DQw1 and particularly of DRw6-DQw1 haplotypes has been reported in these patients [34]. Therefore, the cause of REM sleep dysregulation and narcoleptic-like phenotype in DM1 is still unclear, although the known toxicity of the expanded mRNA might affect the alternative splicing of one or more transcripts other than those of the hypocretin genes [8].

## 10. Daytime Sleepiness and Fatigue in DM2

It has been suggested that daytime sleepiness has a similar prevalence in DM2 as well as in DM1 [38]; however, the real magnitude of sleep dysfunction in DM2 has not yet been clarified. 

In striking contrast with DM1, in which EDS is a critical issue, a recent controlled study based on self-reported questionnaires [13] found that EDS was not a prominent clinical feature of the DM2 population as evaluated by means of subjective instruments. Only 6.9% of DM2 patients had EDS compared with 44.8% of DM1 patients and 6.2% of controls. Poor sleep quality, as evaluated by means of Pittsburgh Sleep Quality Index (PSQI), was evident both in DM2 and DM1, and significantly poorer than in healthy controls. Nocturnal sleep impairment was not explained by psychiatric disorders such as depression or other comorbidities, but it was mainly related to sleep disturbances as a result of nocturnal pain. Severe fatigue was evident in both DM2 and DM1 (66% versus 85% patients). Very recently, a small case series has reported EDS in 6 patients with DM2 and MSLT performed in 4 patients showed reduced MSL without SOREMPs [15]. Thus, daytime drowsiness due to a primary CNS hypersomnia can be seen also in DM2, but it is unclear if this represents a central pathophysiologic mechanism due to multiple brain and/or brainstem damages [66]. 

## 11. Sleep Abnormalities/Disturbances in DM2

Little is known regarding the occurrence of sleep disturbances in DM2. Yet, the first report of poor sleep quality in DM2 was mainly focused on increased sleep latency and sleep disturbances as evaluated by means of PSQI [13]. Pain-induced nocturnal awakenings were the most common symptoms mentioned in the DM2 group [13]. In fact, widespread musculoskeletal pain is a well-known disabling feature of DM2, which has previously been described as exercise-related and multifaceted in character [72], and it may share similar features with fibromyalgia [73]. 

Although pain may be considered a valuable factor influencing poor sleep quality, current data suggest that SDB such as obstructive sleep apnoea, central apnoea, or paradoxical breathing during REM sleep may represent a consistent finding [14, 15]. 

The clinical spectrum of DM2 also include PLMS, RLS, insomnia, and REM without atonia with dream-enacting behavior. The latter finding may be emblematic. RSWA and/or REM behavior disorders may represent a further mechanism of REM sleep dysregulation, suggesting that sleep dysfunction may be similar in both DM2 and DM1 [8, 15, 74]. 

However, the pathogenesis of sleep disturbance in DM2 patients remains conjectural due to a lack of controlled polysomnographic studies. Obstructive sleep apnoea may be due to upper airway muscle weakness and myotonia. As in DM1, an abnormality of central control of breathing and sleep-wakefulness related to cerebral involvement may be responsible for sleep-wake and respiratory dysfunction [66]. Sleep disorders in DM2 are summarised in Table 2.

## 12. Our Experience Regarding Sleep Disorders and DM2 by means of Polysomnographic Data and Self-Reported Questionnaires

**Figure 2:**
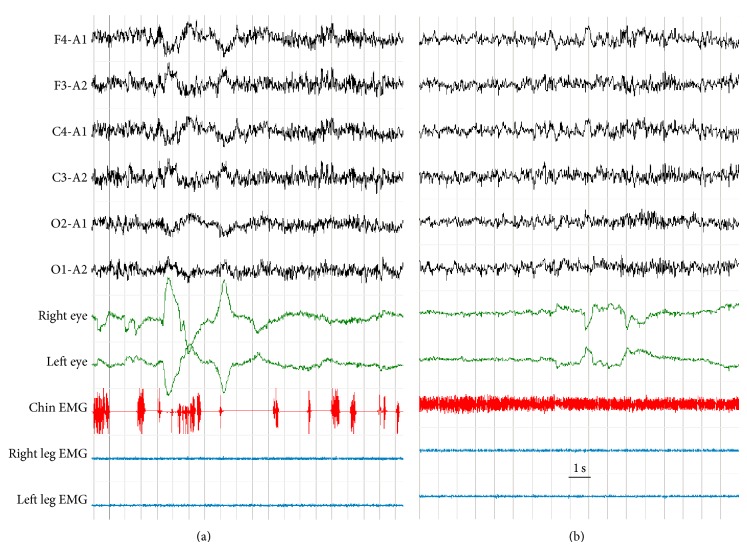
Polysomnograms of a DM2 patient showing REM sleep without atonia with dream-enacting behavior history. Eye leads show rapid movements and EEG channels show desynchronized activity; both are features of REM sleep. (a) Excessive phasic electromyographic activity and intermittent increased tonic electromyographic activity in the chin with normal atonia in the limbs during REM sleep. (b) Sustained tonic electromyographic activity in the chin with normal atonia in the limbs during REM sleep. Right eye and left eye: electrooculogram. F4, F3, C3, C4, O1, and O2: electrode positions according to the 10/20 International System, referenced to combined ears (A1, A2). Chin EMG: electromyography of the mentalis muscle. Left and right leg EMG: electromyography of the left and right tibialis anterior. Note the time calibration mark.

We evaluated a cohort of 12 consecutive patients with genetically determined DM2. Two patients had snoring and one had dream-enacting behavior. Fifty-eight per cent reported pain as the cause of sleep disturbance during the past month and 16% complained of daytime hypersomnolence by means of subjective rating scales. PSG data showed increased arousability and low sleep efficiency (<90%) in all DM2 patients. Seven DM2 patients (58%) were affected by obstructive sleep apnoea, whereas 25% presented PLMS (PLMI > 15/h). RSWA was evident in 50% of DM2 patients, and one of them also reported a history of dream enactment behavior and severe obstructive sleep apnoea syndrome (RDI 49/h) (see Figure 2). Finally, only one patient had few periods of central apnoeas. According to objective data, DM2 patients showed the worst scores on the PSQI components regarding the sleep disturbances and daytime dysfunction. Furthermore, EDS, as shown by an MSL < 8 min at MSLT, was evident in 33% of DM2 patients without SOREMPs, suggesting a natural propensity of DM2 to somnolence [75]. Twenty-five per cent of DM2 subjects (3/12) presented an ESS score > 10, whereas two DM2 patients (16.6%) had a DSS value ≥7. As underlined by Bhat and colleagues, these sleep abnormalities (SDB and RSWA) are novel observations in DM2 and their pathogenesis remains conjectural [15]. Sleep apnoea, due to upper airway muscle weakness and myotonia, myalgic pain, or a direct CNS involvement may account for the sleep fragmentation and daytime sleepiness in DM2 patients. However sleep apnoea may represent a confounding factor, since RSWA may compensate and protect against apnoea episodes as recently theorized [76]. This “compensatory” hypothesis may be confirmed by a higher prevalence of REM sleep impairment in younger DM1 patients with milder hypoxia and lower daytime sleep shown in previous studies [8]. On the other hand, RSWA could be easily related to the brainstem and diencephalon involvement in DM2 [66], particularly at the level of the pedunculopontine and laterodorsal tegmental nuclei that are the critical modulators of activated behavioral states such as wakefulness and REM sleep [77]. Whether sleep dysfunction in DM2 is of the same magnitude as in DM1 cannot be determined without further controlled studies in a larger number of patients. 

## 13. Sleep Dysfunction, Social Participation, and Quality of Life in DM

The presence of sleep disturbances can affect daily life, general wellbeing, and social participation in both DM1 and DM2 patients in comparison to the general population [5, 78–80].

Sleep disorders and daytime sleepiness may account for this impairment of quality of life. These symptoms often interfere with work responsibilities and domestic activities, causing great functional disability in many patients [24, 41]. As underlined by Phillips and colleagues, somnolence scores positively correlated with disability severity scores in DM1 patients [55]. Regarding the quality of life, DM1 hypersomnolent patients report lower scores on all domains of SF36 in comparison with patients without EDS [37, 81].

Although fatigue is not described as the primary symptom of DM, patients report this clinical feature as having the greatest effect on their lives [80, 82]. When daytime sleepiness is marked, it may be associated with falling asleep at work and ultimately prevent some DM1 patients from remaining in employment [41]. Moreover, both fatigue and pain appear to be related to a decrease of health status and vitality in DM2 population [80]. 

## 14. Management and Treatment of Sleep Disturbances in DM

DM patients should be routinely assessed for EDS and sleep disorders in view of the potential deleterious impact of these symptoms on social and physical functioning and quality of life. 

Firstly, physicians should clarify the phenotype of the main sleep complaint by means of a detailed sleep schedule and an accurate clinical interview, always considering DM-related sedentary activities and functional limitations. Moreover the patient's bed partner should be interviewed to assess the presence of nocturnal sleep disturbances and complaint of EDS. In order to quantify the sleep-related problems, physicians may utilize sleep logs, prolonged actigraphic recordings, daytime sleepiness, fatigue, pain and depression rating scales, sleep disorder questionnaire, disability index, and health-related quality of life scales. However, PSG remains the gold standard to identify the nature and severity of sleep disorders, and the MSLT for assessment of daytime sleepiness and of putative REM sleep dysregulation. In particular cases, a prolonged continuous PSG could be necessary to assess the degree of abnormally long sleep time, and the MWT to assess the efficacy of treatment intake. 

Early recognition and treatment of SDB with nocturnal noninvasive mechanical ventilation are mandatory [60]. In absence of diagnosed SDB, a periodical nocturnal cardiorespiratory monitoring should be proposed even in the absence of SDB suggestive symptoms. Ambulatory oximetry recording may first be recommended as a practical screening tool in DM. 

DM patients with severe sleep apnoeas may be treated with various forms of ventilation, intermittent positive pressure breathing, continuous positive airway pressure (CPAP), bilevel positive airway pressure (BiPAP), and the more recently introduced adaptive servoventilation with particular interest for the central apnoea and/or Cheyne-Stokes breathing and complex sleep apnoea management. 

Despite appropriate management of SDB, EDS persists in most DM patients [53]. In such conditions, CNS stimulant drugs, increasingly used to treat EDS in patients with DM, may be required. The recent American Academy of Sleep Medicine (AASM) practice parameters for the treatment of narcolepsy and other hypersomnias of central origin stated that methylphenidate may be effective for treatment of DM-related EDS [83]. In contrast, a Cochrane review on well-designed psychostimulant trials in children and adults affected with DM1 and EDS concluded on the absence of evidence to support its routine use [84] but highlighted some lines of evidence from two studies that modafinil may improve EDS [36, 85].

## 15. Conclusions and Future Outlook

1. Sleep disturbances and excessive daytime somnolence are common and disabling features in DM1, as shown by several uncontrolled and controlled polysomnographic studies, but their pathophysiology is still poorly understood. To our knowledge, insufficient clinical and polysomnographic data are available regarding the occurrence and the real magnitude of sleep disorders in DM2.
2. SDB, resulting in nocturnal hypoxia and hypercapnia, is the most common sleep disorder in both DM1 and DM2.
3. A “central” hypothesis due to intrinsic CNS defect input to particular brain/brainstem structures seems to be the most likely explanation for hypersomnia and/or REM sleep dysregulation (SOREM, RSWA).
4. The presence of sleep dysfunction in patients with neuromuscular disorders is not always obvious because symptoms often develop insidiously and it may be difficult for patients to discern, attributing the daytime sleepiness and fatigue to their underlying neurologic illness.
5. In addition, sleep dysfunction, particularly SDB, is a major cause of morbidity and mortality in patients with neuromuscular diseases. Persistent nocturnal hypoxemia, the end result of SDB from any cause, results in cardiovascular and pulmonary morbidity and mortality from causes such as lethal cardiac arrhythmias, pulmonary hypertension, right heart failure, and propensity to myocardial infarction and stroke.
6. Sleep disturbances cause sleep fragmentation, excessive daytime sleepiness, leading to disability and affecting mood and cognition. In addition, sleep disturbances may be involved as cardiovascular risk factor and sudden death.
7. In particular, more attention should be paid to evaluate the hypothetic autonomic effects of specific sleep disorders (i.e., RLS, PLMS, and RSWA) in order to test the possibility of an increased propensity to autonomic dysfunction and possibly to sudden death in patients affected by myotonic dystrophies and complaining of specific sleep disorders.
8. Finally, early identification of SDB may help optimizing the treatment, improves the quality of life, and prolongs survival in patients with myotonic dystrophy.

We believe that a prospective evaluation and further trials in large samples of both DM1 and DM2 patients are necessary to clarify the real magnitude of sleep dysfunction and for optimally treating patients affected by this progressive neurodegenerative condition. These studies are strongly needed particularly in DM2 patients, also considering recent evidences showing that DM2 might be more frequent than DM1 in some countries (i.e., Finland, Germany) and largely undiagnosed [86, 87].

## Figures and Tables

**Table 1:** Sleep disorders and DM1.

Sleep disorder	Sleep findings	References
SDB	OSA CSA OSAS & CSA-CSR	Romigi et al., 2011 [10]; Yu et al., 2011 [9]; Cirignotta et al., 1987 [29] Pincherle et al., 2012 [56]

Idiopathic hypersomnia	LowCSF orexin A Normal CSF Orexin A Loss of 5HT neurons of DRN EDS & HLA DRW6-DQW1	Martinez-Rodríguez et al., 2003 [13] Ciafaloni et al., 2008 [32] Ono et al., 1998 [12] Manni et al., 1991 [33]

Restless legs syndrome & PLMS	PLMS > 5/h in 38% childhood-onset DM1 uncontrolled PSG study PLMS > 5/h in 61.1% adult-onset DM1 (controlled) PLMW/PLMS	Quera Salva, 2006 [20] Romigi et al., 2011 [10] Ciafaloni et al. 2008 [32]; Yu et al., 2011 [9]

REM sleep dysregulation	Narcoleptic-likephenotype	Martinez-Rodríguez et al., 2003 [13]
	SOREMPs and low MSL (MSLT)	Martinez-Rodríguez et al., 2003 [13]; Ciafaloni et al., 2008 [32]; Laberge et al., 2009 [14, 27]; Yu et al., 2011 [9];
	Higher REM density	Yu et al, 2011 [9]

OSA: obstructive sleep apnea; CSA: central sleep apnea; CSA-CSR: central sleep apnea and cheyne_stokes respiration; CSF: cerebrospinal fluid; 5HT serotonine; DRN: dorsal raphe nucleus; EDS: excessive daytime somnolence; PLMS: periodic limb movement of sleep; PLMW: periodic limb movement of wake; SOREMPs: sleep onset REM periods.

**Table 2:** Sleep disorders and myotonic dystrophy type 2.

Authors	Sleep findings	Number of patients	Methodology
Shepard et al., 2012 [17]	EDS (6/8, 75%) Insomnia (5/8, 62.5%) excessive fatigue (4/8 50%) OSA (3/5 60%) RLS (4/8, 50%)	8 pts (5/8 PSG)	Retrospective uncontrolled PSG study in selected DM2 patients sleep complaints

Bhat et al., 2012 [18]	EDS (6/6, 100%; 4/6 low MSL 66%, no SOREMPs) Insomnia (2/6, 33.3%) Snoring (4/6 66.6%) OSA (4/6 66.6%) REM without atonia with dream enacting behavior in OSAS after CPAP (1/6)∗ Paradoxical breathing in REM (2/6 33.3%) RSWA (1/6, 16.6%) Low sleep efficiency, alfa-delta sleep (2/6 33%)	6 pts (6/6 PSG)	Prospective uncontrolled PSG study in selected DM2 patients sleep complaints

Chokroverty et al., 2012 [57]	REM without atonia with dream enacting behavior in OSAS after CPAP∗	Single Case report	Video-PSG

Tieleman et al., 2010 [16]	EDS 6.9% (DM2) 44.8% (DM1) 6.2% (Control) Poor sleep quality 66% (DM2) 45% (DM1) 26% (Control)	29 DM2, 29 DM1, 65 Controls	Prospective controlled study with subjective scales

Romigi et al., 2013 [75]	Sleep disturbance pain-related (PSQI) (7/12 58%) EDS (subjective scales 2/12, 66.6%; MSLT 4/12 33% no SOREMPs) Snoring (2/12 16.6%) Low sleep efficiency (PSG 100% DM2) OSA (7/12 58%) PLMS (PLMI > 15/h 3/12 25%) RSWA with dream enacting behavior in severe OSAS (8.3% 1/12) Paradoxical breathing in REM (1/12 8.3%) RSWA (6/12, 50%)	12 DM2, 18 DM1, and 12 Controls	Prospective controlled study with PSG and subjective scales

EDS: excessive daytime somnolence; OSA: obstructive sleep apnea; CSA: central sleep apnea; PLMS: periodic limb movement of sleep; PLMW: periodic limb movement of wake; SOREMPs: sleep onset REM periods; RSWA: REM sleep without atonia

∗Probably the same patient.

## Abbreviations

AASM: American Academy of Sleep Medicine
AHI: Apnoea-hypopnoea index
BMI: Body mass index
CSF: Cerebrospinal fluid
DM1: Myotonic dystrophy type 1
DM2: Myotonic dystrophy type 2
DSS: Daytime sleepiness scale
EDS: Excessive daytime somnolence
ESS: Epworth sleepiness scale
FDSS: Rasch-built fatigue & daytime sleepiness scale
GH: Growth hormone
HPA: Hypothalamic-pituitary-adrenal
KFSS: Krupp's fatigue severity scale
MSL: Mean sleep latency
MSLT: Multiple sleep latency test
MWT: Maintenance of wakefulness test
PLMS: Periodic limb movement of sleep
PLMW: Periodic limb movement of wake
PSG: Polysomnography
PSQI: Pittsburgh sleep quality index
REM: Rapid eye movement
RSWA: REM sleep without atonia
SDB: Sleep disordered breathing
SOREMPs: Sleep-onset rapid eye movement periods
WASO: Wakefulness after sleep onset.

## References

[1] Turner C., Hilton-Jones D. (2010). The myotonic dystrophies: diagnosis and management. *Journal of Neurology, Neurosurgery and Psychiatry*.

[2] Day J. W., Ricker K., Jacobsen J. F. (2003). Myotonic dystrophy type 2: molecular, diagnostic and clinical spectrum. *Neurology*.

[3] Meola G. (2000). Clinical and genetic heterogeneity in myotonic dystrophies. *Muscle & Nerve*.

[4] Pisani V., Panico M. B., Terracciano C. (2008). Preferential central nucleation of type 2 myofibers is an invariable feature of myotonic dystrophy type 2. *Muscle & Nerve*.

[5] Laberge L., Gagnon C., Dauvilliers Y. (2013). Daytime sleepiness and myotonic dystrophy. *Current Neurology and Neuroscience Reports*.

[6] Yu H., Laberge L., Jaussent I. (2011). Daytime sleepiness and REM sleep characteristics in myotonic dystrophy: a case-control study. *Sleep*.

[7] Romigi A., Izzi F., Pisani V. (2011). Sleep disorders in adult-onset myotonic dystrophy type 1: a controlled polysomnographic study. *European Journal of Neurology*.

[8] Dauvilliers Y. A., Laberge L. (2012). Myotonic dystrophy type 1, daytime sleepiness and REM sleep dysregulation. *Sleep Medicine Reviews*.

[9] Ono S., Takahashi K., Jinnai K. (1998). Loss of serotonin-containing neurons in the raphe of patients with myotonic dystrophy: a quantitative immunohistochemical study and relation to hypersomnia. *Neurology*.

[10] Martinez-Rodríguez J. E., Lin L., Iranzo A. (2003). Decreased hypocretin-1 (orexin-A) levels in the cerebrospinal fluid of patients with myotonic dystrophy and excessive daytime sleepiness. *Sleep*.

[11] Laberge L., Bégin P., Dauvilliers Y. (2009). A polysomnographic study of daytime sleepiness in myotonic dystrophy type 1. *Journal of Neurology, Neurosurgery and Psychiatry*.

[12] Massa R., Bucci E., Bianchi M. (2012). A roman network for the myotonic dystrophies: start-up and construction of a patients’database. *Neurological Sciences*.

[13] Tieleman A. A., Knoop H., van de Logt A., Bleijenberg G., van Engelen B. G. M., Overeem S. (2010). Poor sleep quality and fatigue but no excessive daytime sleepiness in myotonic dystrophy type 2. *Journal of Neurology, Neurosurgery and Psychiatry*.

[14] Shepard P., Lam E. M., St Louis E. K. (2012). Sleep disturbances in myotonic dystrophy type 2. *European Neurology*.

[15] Bhat S., Gupta D., Chokroverty S. (2012). Sleep disorders in neuromuscular diseases. *Neurologic Clinics*.

[16] Liquori C. L., Ricker K., Moseley M. L. (2001). Myotonic dystrophy type 2 caused by a CCTG expansion in intron I of ZNF9. *Science*.

[17] Massa R., Panico M. B., Caldarola S. (2010). The myotonic dystrophy type 2 (DM2) gene product zinc finger protein 9 (ZNF9) is associated with sarcomeres and normally localized in DM2 patients' muscles. *Neuropathology and Applied Neurobiology*.

[18] Udd B., Krahe R. (2012). The myotonic dystrophies: molecular, clinical, and therapeutic challenges. *The Lancet Neurology*.

[19] Botta A., Bonifazi E., Vallo L. (2006). Italian guidelines for molecular analysis in myotonic dystrophies. *Acta Myologica*.

[20] Hilton-Jones D. (1997). Myotonic dystrophy—forgotten aspects of an often neglected condition. *Current Opinion in Neurology*.

[21] Quera Salva M., Blumen M., Jacquette A. (2006). Sleep disorders in childhood-onset myotonic dystrophy type 1. *Neuromuscular Disorders*.

[22] van der Meche F. G. A., Bogaard J. M., van der Sluys J. C. M., Schimsheimer R. J., Ververs C. C. M., Busch H. F. M. (1994). Daytime sleep in myotonic dystrophy is not caused by sleep apnoea. *Journal of Neurology Neurosurgery and Psychiatry*.

[23] Park Y. D., Radtke R. A. (1995). Hypersomnolence in myotonic dystrophy: demonstration of sleep onset REM sleep. *Journal of Neurology Neurosurgery and Psychiatry*.

[24] Rubinsztein J. S., Rubinsztein D. C., Goodburn S., Holland A. J. (1998). Apathy and hypersomnia are common features of myotonic dystrophy. *Journal of Neurology Neurosurgery and Psychiatry*.

[25] Phemister J. C., Small J. M. (1961). Hypersomnia in dystrophia myotonica. *Journal of Neurology, Neurosurgery, and Psychiatry*.

[26] Goldenberg F. L., Perrier M., Duizabo D. P. (1977). Disturbances of alertness, sleep and respiratory function in dystrophia myotonica. *Revue Neurologique*.

[27] Harper P. S. (2001). *Miotonic Dystrophy*.

[28] Laberge L., Dauvilliers Y., Bégin P., Richer L., Jean S., Mathieu J. (2009). Fatigue and daytime sleepiness in patients with myotonic dystrophy type 1: to lump or split?. *Neuromuscular Disorders*.

[29] Frens P. D. H. (1981). Hypersomnia associated with alveolar hypoventilation in myotonic dystrophy. *Neurology*.

[30] Cirignotta F., Mondini S., Zucconi M. (1987). Sleep-related breathing impairment in myotonic dystrophy. *Journal of Neurology*.

[31] Gibbs J. W., Ciafaloni E., Radtke R. A. (2002). Excessive daytime somnolence and increased rapid eye movement pressure in myotonic dystrophy. *Sleep*.

[32] Laberge L., Bégin P., Montplaisir J., Mathieu J. (2004). Sleep complaints in patients with myotonic dystrophy. *Journal of Sleep Research*.

[33] Ciafaloni E., Mignot E., Sansone V. (2008). The hypocretin neurotransmission system in myotonic dystrophy type 1. *Neurology*.

[34] Manni R., Zucca C., Martinetti M., Ottolini A., Lanzi G., Tartara A. (1991). Hypersomnia in dystrophia myotonica: a neurophysiological and immunogenetic study. *Acta Neurologica Scandinavica*.

[35] Dauvilliers Y., Baumann C. R., Carlander B. (2003). CSF hypocretin-1 levels in narcolepsy, Kleine-Levin syndrome, and other hypersomnias and neurological conditions. *Journal of Neurology, Neurosurgery and Psychiatry*.

[36] Talbot K., Stradling J., Crosby J., Hilton-Jones D. (2003). Reduction in excess daytime sleepiness by modafinil in patients with myotonic dystrophy. *Neuromuscular Disorders*.

[37] Laberge L., Dauvilliers Y., Thorpy M., Billiard M. (2011). Myotonic dystrophy and sleepiness. *Sleepiness: Causes, Consequences, Disorders and Treatment*.

[38] Meola G., Sansone V., Perani D. (2003). Executive dysfunction and avoidant personality trait in myotonic dystrophy type 1 (DM-1) and in proximal myotonic myopathy (PROMM/DM-2). *Neuromuscular Disorders*.

[39] Winblad S., Lindberg C., Hansen S. (2005). Temperament and character in patients with classical myotonic dystrophy type 1 (DM-1). *Neuromuscular Disorders*.

[40] Laberge L., Prévost C., Perron M. (2010). Clinical and genetic knowledge and attitudes of patients with myotonic dystrophy type 1. *Public Health Genomics*.

[41] Hilton-Jones D., Bowler M., Lochmueller H. (2012). Modafinil for excessive daytime sleepiness in myotonic dystrophy type 1—the patients' perspective. *Neuromuscular Disorders*.

[42] Kalkman J. S., Schillings M. L., van der Werf S. P. (2005). Experienced fatigue in facioscapulohumeral dystrophy, myotonic dystrophy, and HMSN-I. *Journal of Neurology, Neurosurgery and Psychiatry*.

[43] Hilton-Jones D., Damian M. S., Meola G., Harper P., van Engelen B., Eymard B., Wilcox D. (2004). Somnolence and its management. *Myotonic Dystrophy: Present Management, Future Therapy*.

[44] Carskadon M. A., Dement W. C., Mitler M. M. (1986). Guidelines for the multiple sleep latency test (MSLT): a standard measure of sleepiness. *Sleep*.

[45] Bonnet M. H., Arand D. L. (2005). Impact of motivation on multiple sleep latency test and maintenance of wakefulness test measurements. *Journal of Clinical Sleep Medicine*.

[46] Bennett L. S., Stradling J. R., Davies R. J. O. (1997). A behavioural test to assess daytime sleepiness in obstructive sleep apnoea. *Journal of Sleep Research*.

[47] Laberge L., Lacroix G., Bégin P., Beaudry M., Mathieu J. (2004). Psycomotor vigilance performance is associated with objective daytime sleepiness in miotonic dystrophy. *Journal of Sleep Research*.

[48] Giubilei F., Antonini G., Bastianello S. (1999). Excessive daytime sleepiness in myotonic dystrophy. *Journal of the Neurological Sciences*.

[49] Cluydts R., De Valck E., Verstraeten E., Theys P. (2002). Daytime sleepiness and its evaluation. *Sleep Medicine Reviews*.

[50] Laberge L., Gagnon C., Jean S., Mathieu J. (2005). Fatigue and daytime sleepiness rating scales in myotonic dystrophy: a study of reliability. *Journal of Neurology, Neurosurgery and Psychiatry*.

[51] Hermans M. C., Merkies I. S., Laberge L. (2013). Fatigue and daytime sleepiness scale in myotonic dystrophy type 1. *Muscle & Nerve*.

[52] Gilmartin J. J., Cooper B. G., Griffiths C. J. (1991). Breathing during sleep in patients with myotonic dystrophy and non-myotonic respiratory muscle weakness. *Quarterly Journal of Medicine*.

[53] Guilleminault C., Philip P., Robinson A. (1998). Sleep and neuromuscular disease: bilevel positive airway pressure by nasal mask as a treatment for sleep disordered breathing in patients with neuromuscular disease. *Journal of Neurology Neurosurgery and Psychiatry*.

[54] van Hilten J. J., Kerkhof G. A., van Dijk J. G., Dunnewold R., Wintzen A. R. (1993). Disruption of sleep-wake rhythmicity and daytime sleepiness in myotonic dystrophy. *Journal of the Neurological Sciences*.

[55] Phillips M. F., Steer H. M., Soldan J. R., Wiles C. M., Harper P. S. (1999). Daytime somnolence in myotonic dystrophy. *Journal of Neurology*.

[56] Coccagna G., Martinelli P., Lugaresi E. (1982). Sleep and alveolar hypoventilation in myotonic dystrophy. *Acta Neurologica Belgica*.

[57] di Costanzo A., Santoro L., de Cristofaro M., Manganelli F., di Salle F., Tedeschi G. (2008). Familial aggregation of white matter lesions in myotonic dystrophy type 1. *Neuromuscular Disorders*.

[58] Hernàndez-Hernàndez O., Guiraud-Dogan C., Sicot G. (2013). Myotonic dystrophy CTG expansion affects synaptic vesicle proteins, neurotransmission and mouse behavior. *Brain*.

[59] Broughton R., Stuss D., Kates M., Roberts J., Dunham W. (1990). Neuropsychological deficits and sleep in myotonic dystrophy. *Canadian Journal of Neurological Sciences*.

[60] Pincherle A., Patruno V., Raimondi P. (2012). Sleep breathing disorders in 40 Italian patients with myotonic dystrophy type 1. *Neuromuscular Disorders*.

[61] Kiyan E., Okumus G., Cuhadaroglu C., Deymeer F. (2010). Sleep apnea in adult myotonic dystrophy patients who have no excessive daytime sleepiness. *Sleep and Breathing*.

[62] Ono S., Kanda F., Takahashi K. (1996). Neuronal loss in the medullary reticular formation in myotonic dystrophy: a clinicopathological study. *Neurology*.

[63] Meola G., Sansone V. (2007). Cerebral involvement in myotonic dystrophies. *Muscle & Nerve*.

[64] Pennestri M. H., Montplaisir J., Colombo R., Lavigne G., Lanfranchi P. A. (2007). Nocturnal blood pressure changes in patients with restless legs syndrome. *Neurology*.

[65] Walters A. S., Rye D. B. (2010). Evidence continues to mount on the relationship of restless legs syndrome/periodic limb movements in sleep to hypertension, cardiovascular disease, and stroke. *Sleep*.

[66] Minnerop M., Weber B., Schoene-Bake J. (2011). The brain in myotonic dystrophy 1 and 2: evidence for a predominant white matter disease. *Brain*.

[67] Ono S., Kurisaki H., Sakuma A., Nagao K. (1995). Myotonic dystrophy with alveolar hypoventilation and hypersomnia: a clinicopathological study. *Journal of the Neurological Sciences*.

[68] Culebras A., Podolsky S., Leopold N. A. (1977). Absence of sleep related growth hormone elevations in myotonic dystrophy. *Neurology*.

[69] Johansson Å., Carlström K., Ahrén B. (2000). Abnormal cytokine and adrenocortical hormone regulation in myotonic dystrophy. *The Journal of Clinical Endocrinology and Metabolism*.

[70] Ashizawa T. (1998). Myotonic dystrophy as a brain disorder. *Archives of Neurology*.

[71] Romeo V., Pegoraro E., Ferrati C. (2010). Brain involvement in myotonic dystrophies: neuroimaging and neuropsychological comparative study in DM1 and DM2. *Journal of Neurology*.

[72] George A., Schneider-Gold C., Zier S., Reiners K., Sommer C. (2004). Musculoskeletal pain in patients with myotonic dystrophy type 2. *Archives of Neurology*.

[73] Auvinen S., Suominen T., Hannonen P., Bachinski L. L., Krahe R., Udd B. (2008). Myotonic dystrophy type 2 found in two of sixty-three persons diagnosed as having fibromyalgia. *Arthritis and Rheumatism*.

[74] Chokroverty S., Bhat S., Rosen D., Farheen A. (2012). REM behavior disorder in myotonic dystrophy type 2. *Neurology*.

[75] Romigi A., Albanese M., Placidi F. (2013). Sleep disorders in myotonic dystrophy type 2: a controlled polysomnographic study and self-reported questionnaires. *European Journal of Neurology*.

[76] Huang J., Zhang J., Lam S. P. (2011). Amelioration of obstructive sleep apnea in REM sleep behavior disorder: implications for the neuromuscular control of OSA. *Sleep*.

[77] Rye D. B. (1997). Contributions of the pedunculopontine region to normal and altered REM sleep. *Sleep*.

[78] Gagnon C., Mathieu J., Noreau L. (2007). Life habits in myotonic dystrophy type 1. *Journal of Rehabilitation Medicine*.

[79] Kierkegaard M., Harms-Ringdahl K., Holmqvist L. W., Tollbäck A. (2009). Perceived functioning and disability in adults with myotonic dystrophy type 1: a survey according to the international classification of functioning, disability and health. *Journal of Rehabilitation Medicine*.

[80] Tieleman A. A., Jenks K. M., Kalkman J. S., Borm G., van Engelen B. G. M. (2011). High disease impact of myotonic dystrophy type 2 on physical and mental functioning. *Journal of Neurology*.

[81] Sansone V. A., Ricci C., Montanari M., Apolone G., Rose M., Meola G. (2012). Measuring quality of life impairment in skeletal muscle channelopathies. *European Journal of Neurology*.

[82] Heatwole C., Bode R., Johnson N. (2012). Patient-reported impact of symptoms in myotonic dystrophy type 1 (PRISM-1). *Neurology*.

[83] Morgenthaler T. I., Kapur V. K., Brown T. (2007). Practice parameters for the treatment of narcolepsy and other hypersomnias of central origin: an American Academy of Sleep Medicine report. *Sleep*.

[84] Annane D., Moore D. H., Barnes P. R., Miller R. G. (2006). Psychostimulants for hypersomnia (excessive daytime sleepiness) in myotonic dystrophy. *Cochrane Database of Systematic Reviews*.

[85] MacDonald J. R., Hill J. D., Tarnopolsky M. A. (2002). Modafinil reduces excessive somnolence and enhances mood in patients with myotonic dystrophy. *Neurology*.

[86] Bachinski L. L., Udd B., Meola G. (2003). Confirmation of the type 2 myotonic dystrophy (CCTG)n expansion mutation in patients with proximal myotonic myopathy/proximal myotonic dystrophy of different European origins: a single shared haplotype indicates an ancestral founder effect. *American Journal of Human Genetics*.

[87] Suominen T., Bachinski L. L., Auvinen S. (2011). Population frequency of myotonic dystrophy: higher than expected frequency of myotonic dystrophy type 2 (DM2) mutation in Finland. *European Journal of Human Genetics*.

